# Effect of resistance training on muscle properties and function in women with generalized joint hypermobility: a single-blind pragmatic randomized controlled trial

**DOI:** 10.1186/s13102-021-00238-8

**Published:** 2021-02-08

**Authors:** Gere Luder, Daniel Aeberli, Christine Mueller Mebes, Bettina Haupt-Bertschy, Jean-Pierre Baeyens, Martin L. Verra

**Affiliations:** 1 Department of Physiotherapy, Bern University Hospital, Insel Group, CH-3010 Bern, Switzerland; 2 Faculty of Physical Education and Physical Therapy, Vrije Universiteit Brussel, Pleinlaan 2, 1050 Brussels, Belgium; 3 Department of Rheumatology, Clinical Immunology and Allergology, Bern University Hospital and University of Bern, CH-3010 Bern, Switzerland

**Keywords:** Muscle strength, Exercise therapy, Joint instability, Quality of life

## Abstract

**Background:**

Generalized joint hypermobility is defined as an excessive range of motion in several joints. Having joint hypermobility is not a pathology, but when associated with pain and other symptoms, it might affect health and function. Evidence for physiotherapy management is sparse and resistance training might be a possible intervention. Thus, the effects of 12-week resistance-training on muscle properties and function in women with generalized joint hypermobility were evaluated.

**Methods:**

In this single-blind randomized controlled trial women between 20 and 40 years with generalized joint hypermobility (Beighton score at least 6/9) were included. Participants were randomly allocated to 12-week resistance training twice weekly (experimental) or no lifestyle change (control). Resistance training focused on leg and trunk muscles. Primary outcome was muscle strength; additional outcomes included muscle properties, like muscle mass and density, functional activities, pain and disability. Training adherence and adverse events were recorded.

**Results:**

Of 51 participating women 27 were randomised to training and 24 into the control group. In each group 11 women had joint hypermobility syndrome, fulfilling the Brighton criteria, while 24 (89%) in the training group and 21 (88%) in the control group mentioned any pain. The mean strength of knee extensors varied in the training group from 0.63 (sd 0.16) N/bm before training to 0.64 (sd 0.17) N/bm after training and in the control group from 0.53 (sd 0.14) N/bm to 0.54 (sd 0.15) N/bm. For this and all other outcome measures, no significant differences between the groups due to the intervention were found, with many variables showing high standard deviations. Adherence to the training was good with 63% of participants performing more than 80% of sessions. One adverse event occurred during training, which was not clearly associated to the training. Four participants had to stop the training early.

**Conclusions:**

No improvement in strength or muscle mass by self-guided resistance training was found. Low resistance levels, as well as the choice of outcome measures were possible reasons. A more individualized and better guided training might be important. However, program adherence was good with few side effects or problems triggered by the resistance training.

**Trial registration:**

This trial was prospectively registered in the ISRCTN registry (www.isrctn.com, BMC, Springer Nature) on July 16, 2013 as ISRCTN90224545. The first participant was enrolled at October 25, 2013.

**Supplementary Information:**

The online version contains supplementary material available at 10.1186/s13102-021-00238-8.

## Background

Generalized joint hypermobility (GJH) is defined by a range of motion exceeding the normal limits in several joints. It is usually assessed by the 9 point Beighton score, testing for excessive mobility in the fingers, elbow, knee and lower back [1]. GJH has been found in about 10–30% of all persons, depending on the exact definition [2–4], e.g. Scheper et al. [3] described 22.8% at the cut-off at 4/9 points and 8.8% at 6/9 points in young students. In general, women are more often hypermobile than men, as described by Scheper et al. in 2015 who found 31.9% of women vs. 9.7% of to be generally hypermobile, based on a cut-off at 4/9 points and 13.9% for women vs. 1.5% for men at a cut-off at 6/9 points. Generally there is a decrease of joint mobility with ageing, as illustrated by lower cut-offs used in older persons [4].

By definition, GJH is not necessarily a clinical diagnosis. Numerous persons with GJH do not manifest symptoms and for some sports or in dance it might even be an asset to have extensive mobility [5, 6]. In contrast, having increased joint mobility might result in a wide variety of clinical symptoms [7]. For a long time hypermobile persons with symptoms were diagnosed as having joint hypermobility syndrome (JHS), using the Brighton criteria [8]. They mainly encompassed musculoskeletal complications, but also signs of skin, eye or organ involvement. After years of discussion whether JHS and the hypermobile type of Ehlers-Danlos syndrome (EDS) were the same entity, a new nosology for the EDS was developed in 2017 [9, 10]. As part of this process the definitions and classifications for the spectrum of disorders associated with GJH were revised [7]. The term JHS was discarded and as a new diagnosis, the hypermobility spectrum disorder (HSD) was introduced. Thus, persons with GJH and various symptoms that do not fulfil the new formal criteria for hypermobile Ehlers-Danlos syndrome (hEDS) can now be diagnosed as having HSD.

Nevertheless, having GJH can lead to problems in activities of daily life and is sometimes associated with various impairments and musculoskeletal disorders. Scheper et al. [11] stated that persons with GJH experience more pain, fatigue and disability than controls. In two other reviews was shown that people with GJH have a higher prevalence and incidence of lower limb injuries [12, 13]. A large population study in Denmark found that persons with GJH were more likely to experience knee or shoulder pain and it was up to four times more likely that they avoided some activities due to symptoms [14, 15]. Thus, in the context of prevention it might be important for persons having GJH to stay active to maintain their ability to perform daily life and work-related activities. Additionally, there is a need to find ways to prevent joint pain, disability and possible long-term consequences of the condition.

In terms of interventions, a limited number of studies have been published so far. The review by Scheper et al. [11] found no studies assessing treatments in GJH and only five looking at treatments in persons with JHS, resulting in small effects on pain and inconsistent effects on disability. Comparing persons with GJH to those with normal joint mobility raised several issues, e.g. in a study with 328 adults those with GJH had less strength in the knee, hip, shoulder and forearm and they performed less physical activity [3]. Our previous study with 195 participants presented changes in neuromuscular control during gait and stair climbing [16, 17] as well as in strength, balance and passive tibial translation [18, 19]. People with GJH thus have neuromusculoskeletal impairments, particularly strength deficits, which may make them more susceptible to developing symptoms. It is important to investigate whether such deficits can be improved through preventive rehabilitation.

In physiotherapy, resistance training is well established as an intervention to improve strength and muscle mass, as well as to gain function and decrease impairments [20]. Also, for apparently healthy persons regular exercise is generally recommended and resistance training is an important part in the prevention of diseases and injuries [21]. There is a lack of high quality trials looking at the effects of resistance training in persons with various specific health problems. However, resistance training is regularly prescribed in musculoskeletal physiotherapy and performed by patients with conditions such as low back pain or with osteoarthritis of the hip or knee [20, 22].

Based on the described neuromuscular deficits in persons with GJH the performance of resistance training to gain more strength and muscle mass might help to improve their performance in daily life and to prevent pain, disability and injuries, mainly joint distortions. Not only will the additional muscle strength support the dynamic stabilisation of the joints, but by the strength training also an increase in muscle and tendon stiffness is described, which might also provide more passive support for the joints [23, 24]. Finally, an increase in strength and muscle mass might also improve the impaired proprioception of persons with GJH around the joint and thus provide better joint stabilisation during activities [25, 26].

In this context, the present study was designed to evaluate a guided resistance-training program for women with GJH with or without symptoms. The progressive resistance-training program focussed on increasing muscle mass and strength of leg muscles and the trunk. The primary objective was to measure the immediate effects of this 12 week graded resistance-training program on muscle strength and muscle properties, compared to a control group without training. Secondary aims were to evaluate the impact of the training program on function, pain and disability in women with GJH and to evaluate the feasibility of the training program in terms of adherence and side effects.

## Methods

### Study design

**Fig. 1:**
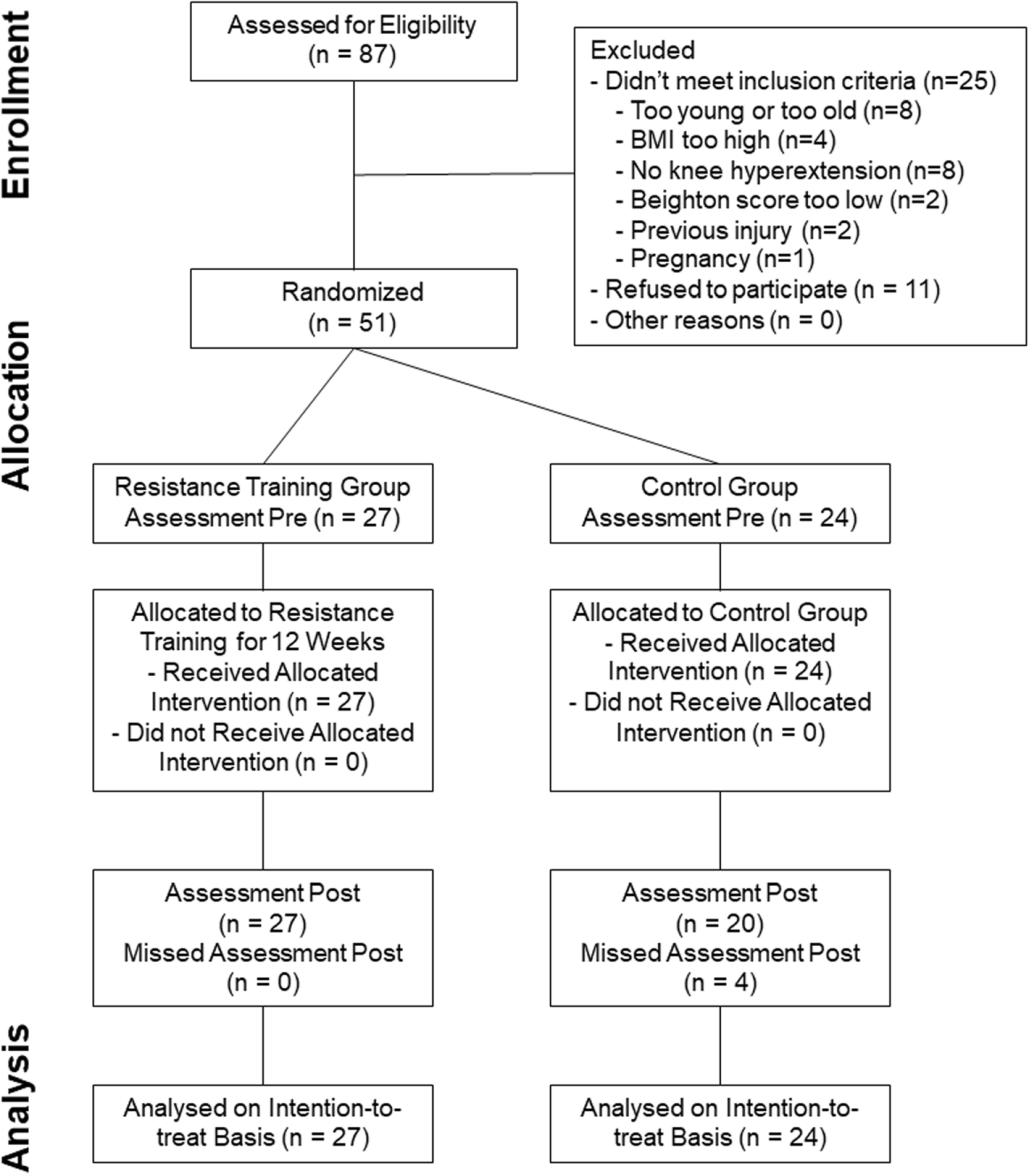
Study Flowchart (according to CONSORT)

This study was designed as an assessor-blinded pragmatic randomised controlled trial (RCT). The participants were randomly allocated to either a 12-week mainly self-guided resistance-training program or a control group that did not change their usual lifestyle habits (Fig. 1). The trial was prospectively registered as ISRCTN90224545 (www.isrctn.com, BMC, Springer Nature) and ethical approval was obtained by the Ethics Committee of Canton Berne, Switzerland (No. 222/12). All participants gave written informed consent and the study was conducted according to the Declaration of Helsinki. This paper follows the CONSORT statement [27] and the intervention is described according to the TIDieR checklist [28].

### Participants

Women aged between 20 and 40 years with GJH were eligible for the study if they scored at least 6/9 points on the Beighton score, and right knee hyperextension was mandatory. The higher cut-off was chosen based on more recent publications [29, 30] and knee hyperextension was required because training focused on the lower limb and assessments were performed mainly on the right side. As further inclusion criteria, participants needed to have a body mass index between 18 and 30 kg/m^2^ and be able to understand German questionnaires.

Excluded were women who had had surgery of the lower extremities or lumbar spine in the last two years, because this might affect their current condition and the ability to perform strength training. In addition, women with acute pain in the back or lower extremities were excluded. Women who regularly undertook more than four hours per week of sport activities were excluded to ensure better homogeneity of the groups in terms of muscle strength and training experience. Pregnant women and those less than one year after delivery were excluded, since changes in the hormonal state may affect the outcome of strength training [31]. Finally, women with known inherited diseases of the connective tissue, mainly Marfan syndrome and Ehlers-Danlos syndromes except hypermobility type and Osteogenesis imperfecta, were excluded. A formal diagnosis of Ehler-Danlos syndrome, hypermobility type, was not a reason for exclusion. Note that the criteria for this study were defined in 2012 and thus not based on the new 2017 nosology for EDS and HSD [7, 10].

### Recruitment, inclusion and allocation

Participants were mainly recruited from an existing database of previous studies [17, 32] and via the staff of Bern University Hospital and students of the Bern University of Applied Sciences, Department of Health, Switzerland. Furthermore, announcements in the local newspapers were published to recruit participants. The recruitment period was between August 2013 and November 2015 and the recruitment, as well as all the measurements and training sessions took place at Bern University Hospital, in Bern, Switzerland.

Interested participants were informed by phone before their first appointment and received information sheets by mail. After signing the informed consent, inclusion and exclusion criteria were confirmed face-to-face by one physiotherapist (CM), with more than 12 years of clinical experience. The participants performed a standard pregnancy test themselves using a urine sample. For the Beighton score the test movements were a.) Hyperextension of elbow more than 10°, b.) Hyperextension of knee more than 10°, c.) Ability to touch the floor with the palms of the hands, keeping the knees fully extended, d.) At least 90° dorsiflexion of 5th metacarpophalangeal joint, and e.) Ability to touch the inner side of the forearm with the thumb [1]. All items, except c.), were tested bilaterally, resulting in a possible total score of 9 points.

The range of motion of the right knee in flexion and extension was measured with a standard inclinometer while lying supine. Additional measures included body weight, body height, arm span, and arm and leg length on both sides. Finally, anamnestic checking of the Brighton criteria [8] was done by semi-structured interview by the same experienced physiotherapist (CM). The Brighton criteria were recorded for a clearer description of the study group and to allow for potential analysis of the effects or the feasibility of the training for women with and without JHS.

After inclusion, the participants were randomised based on an independently computer-generated randomisation list either to the resistance training or to the control group. After recording of the personal and anamnestic data in the database and confirming inclusion, the physiotherapist responsible for the inclusion accessed the allocation electronically to ensure concealment. The randomisation list was kept secret from the assessor and statistician until all analyses had been performed.

### Intervention

The intervention for the training group was a mainly self-guided 12-week resistance training program to address hypertrophy, focusing on the muscles of the lower extremities and the trunk. Two training sessions of about 50 min were performed each week in the medical training centre of the Berne University Hospital, resulting in 24 training sessions.

The strength training program was developed based on recommendations of the American College of Sports Medicine [21, 33]. The details of the training program are provided in a supporting information file (Luder-G_S1-file_training-program-intervention.pdf). Resistance was mainly set at 80% of the one repetition maximum and three series with 12 repetitions for each side were performed. Four experienced physiotherapists gave the instructions on a 1:1 basis for the training program. All of them regularly instructed patients and healthy persons in the medical fitness and were specifically instructed for this project. In the first week, a one-hour session was dedicated to basic instructions and determining the one repetition maximum. In week three a half-hour session aimed to reassess exercise performance and adapt the resistance. Finally, in week six an additional half-hour session was spent monitoring the proper practice. All other training sessions were performed individually and not directly supervised; however, a responsible physiotherapist was always available in the training room for questions and support. Participants were encouraged to increase the resistance gradually whenever more than 12 repetitions were possible. If pain or discomfort occurred because of the exercise, the women could always refer to the physiotherapist in charge. During the instruction sessions possible adaptations to pain or muscular problems were discussed and suggested, e.g. reduction of resistance, increased rest time between series or a reduction to one or two training series instead of three.

The participants in the control group were advised not to change their lifestyle habits for the next 12 weeks. After the post-measurement, all participants of the control group were offered to participate in the same structured training program as the intervention group.

### Adherence and problems triggered by training

A secondary aim of the project was to assess the feasibility of the resistance training for women with GJH. Thus, the participants recorded the number of training sessions and the exercises performed with all details in a diary. Additionally, personal notes and experiences were documented, e.g. pain, discomfort, or reasons for reduced performance. Performance of more than 80% of the training sessions was deemed as acceptable adherence.

Furthermore, during the training pain and disability in the daily life of the participants were monitored with a face-validated questionnaire using 5-point Likert-scales. The first two questions asked for details of disability or pain during and after the training. Three additional questions asked for other pain or impairments during the week. For every question, the location of the problems and additional information could be provided. The questionnaire was developed based on a previous study [34].

### Outcome assessments

GJH may affect an individual in several dimensions of life, as defined in the International Classification of Functioning, Disability and Health (ICF) [35]. The outcome assessments in this study aimed to evaluate the effects of resistance training in various dimensions of the ICF: muscle strength and properties as body structures, muscle activity during stair climbing in terms of function; and a set of patient reported questionnaires regarding activities and participation to detect impairments and restrictions in daily life. A detailed description of all assessments and the respective analyses is provided in a supporting information file (Luder-G_S2-file_outcome-measures.pdf).

In brief, muscle strength was measured as maximum isometric contraction and rate of force development of the knee extensors and knee flexors on a custom-built strength table using a strain gauge. For each muscle group three measurements were performed. Maximum strength and rate of force development as the slope of the force curve between 20 and 80% of maximum were calculated, the values normalised to body mass and the best attempt taken for calculations [18]. The muscle properties of the thigh were measured using peripheral quantitative computer tomography (pQCT) and muscle cross sectional area, and muscle mass and density were calculated as previously described [36]. The cross-sectional area parameters were all calculated in relation to body mass.

During stair climbing on a standard six-step stair-case [16, 37] the ground reaction forces were measured by a force plate embedded in the 3rd step. Simultaneously the muscle activity of the vastus medialis, vastus lateralis, semitendinosus and biceps femoris was measured using electromyography (EMG). Electrode placement and measurement procedure were defined according to the recommendations of SENIAM [38]. The participants had to climb up and down the stair ten times at a comfortable, self-selected speed barefoot and without using the handrail. All ground reaction forces, and electromyography data were processed with custom-made software and six trials were selected for the analyses of stair ascent and descent. Dynamic EMG data were normalised to the corresponding 100% maximum voluntary contraction value and peak and mean muscle activation during stance were calculated. The vertical ground reaction force curves were normalised to body mass and standard parameters for force and time were calculated as means of six trials for each condition [37].

To measure general health the widely used Medical Outcomes Study Short Form 36-Item (SF-36) health survey was completed and the scores calculated according to the standard method [39]. As a measure of disability in daily life the Arthritis Impact Measurement Scales 2 (AIMS-2), originally developed for patients with rheumatoid arthritis [40], was used, since there was no specific questionnaire for persons with GJH at the time of the study preparation. All scores were calculated according to the described methods [41]. Additionally, and based on previous studies a face-validated questionnaire for hypermobility (HM-Q) was used, asking for pain at specific sites and disability in selected daily life activities. All items were rated on a five-point Likert scale and the sum score for the whole questionnaire calculated.

All assessments were performed by a single investigator (GL), blinded to group allocation. The first assessment took place before the training or control period and the second within two weeks after the end of training or the 12-week control period of the control group.

### Primary and secondary outcomes

The primary outcome for the effect of resistance training was defined as the increase in muscle strength in relation to body mass, measured as maximum voluntary isometric contraction of the knee flexors and extensors. Secondary outcomes included rate of force development of these knee muscles, the cross-sectional area parameters of the thigh, as well as muscle mass and density. All further variables were analysed in an exploratory manner.

Regarding the feasibility of the training intervention, the percentage of completed training sessions was the main parameter. Additionally, pain and disability in daily life as detected by the weekly questionnaire served as further descriptive outcomes.

### Power estimation

As this was the first trial to investigate a resistance training program in individuals with GJH there were only approximate data available for the power calculation. In a previous study [32] a 16.2% higher normalised rate of force development was found for hypermobile women compared to women with normal mobility. With a similar change induced by the training, a hypothetical medium effect size of about 0.6 could be expected.

Derived from this data, a power estimation was performed using G*Power 3.1.5 [42]. For an estimated effect size of 0.6 with the significance level (α) set at *p* ≤ 0.05, a sample size of 21 in each group (total of 42 subjects) was necessary to achieve a power of 0.8. Since some dropouts were expected, the aim was to enrol 50 women in the study.

### Statistical analysis

All analyses were performed on an intention-to-treat basis and included all randomised participants. Missing data was processed by means of imputation based on linear regression per group, except by “last carry forward” for the HM-Q. Missing data for EMG measurements due to technical reasons was not imputed. All statistics were performed on a blinded basis, whereby the randomisation code was only broken after completion of the statistical evaluation.

Descriptive statistics for all clinically relevant parameters are presented. Normal distribution of the data was checked by the Shapiro-Wilk test and Q-Q-plotting to decide whether parametric or non-parametric tests were used for significance testing. At baseline, the comparability between the groups in terms of demographic and prognostic factors was assessed using the independent t-test.

For parametric testing the primary outcomes of the two groups were compared by a mixed analysis of variance (ANOVA) with time as the within subjects factor and group as a between subject factor. To account for possible baseline differences all prognostic variables with significant t-test at baseline between the two groups were additionally introduced as co-variates in the model (ANCOVA). The significance level was Bonferroni-corrected to account for multiple testing (two primary variables) and set at *p* < 0.025 as the accepted significance level.

For the main parameters, mean differences of change for each group as well as 95% confidence intervals (CI) are presented and the respective effect sizes calculated as partial eta square and converted to Cohens d. The additional outcomes of the secondary analyses were not tested for significance but are reported as descriptive data, with mean difference between pre and post and the respective 95% confidence interval (95% CI). A tendency for a change was noted when the 95% CI for the mean difference did not cross the zero line.

## Results

### Participants

Of 87 women assessed for eligibility 51 participated in the study, as depicted in the flow chart (Fig. 1). 25 women were excluded for various reasons, mainly age, high body mass index, not fulfilling the right knee hypermobility criterion, pregnancy or Beighton score too low. Additionally, 11 women declined participation, mainly due to lack of time for the training. The main characteristics of the participants at baseline are shown in Table 1. No differences between groups were seen in terms of age, height, weight, and body mass index. Despite randomisation, the subjects in the training group showed on average significantly higher values for maximum voluntary contraction of the knee extensors (mean (sd) = 0.53 (0.14) vs 0.63 (0.16) N/bm, *p* = 0.015) and flexors (mean (sd) = 0.34 (0.12) vs 0.26 (0.11) N/bm, *p* = 0.016). Consequently, these two variables were introduced as co-variates in the statistical analysis of the outcomes.

**Table 1:** Group Characteristics at Baseline as Mean (Standard Deviation)

	All Participants	Control Group	Training Group	t-test
	(*n* = 51)	(*n* = 24)	(*n* = 27)	*p*-value
Age [years]	26.5 (4.5)	27.0 (4.9)	26.1 (4.2)	0.520
Height [m]	1.68 (0.06)	1.69 (0.07)	1.67 (0.05)	0.329
Weight [kg]	62.6 (10.1)	62.9 (10.5)	62.3 (9.9)	0.822
BMI [kg/m^2^]	22.1 (2.8)	22.0 (2.9)	22.2 (2.8)	0.786
MVC knee extensors [N/bm]	0.58 (0.16)	0.53 (0.14)	0.63 (0.16)	0.015
RFD knee extensors [N/s/bm]	2.38 (1.26)	2.03 (1.31)	2.70 (1.16)	0.058
MVC knee flexors [N/bm]	0.30 (0.13)	0.26 (0.11)	0.34 (0.12)	0.016
RFD knee flexors [N/s/bm]	1.07 (0.87)	0.87 (0.81)	1.25 (1.90)	0.122
mCSA thigh [mm^2^/bm]	13.1 (1.9)	13.1 (2.1)	13.0 (1.7)	0.919
Muscle mass [mg]	659 (96)	645 (94)	645 (92)	0.994
Beighton score				
6/9 [n (%)]	6 (11.8)	2 (8.3)	4 (14.8)	
7/9 [n (%)]	10 (19.6)	6 (25.0)	4 (14.8)	
8/9 [n (%)]	17 (33.3)	8 (33.3)	9 (33.3)	
9/9 [n (%)]	18 (35.3)	8 (33.3)	10 (37.0)	
Brighton criteria yes [n (%)]	22 (43.1)	11 (45.8)	11 (40.7)	
GJH + pain [n (%)]	47 (92.2)	21 (87.5)	24 (88.8)	

*BMI* body mass index, *MVC* maximum voluntary contraction strength, *RFD* rate of force development, *mCSA* muscle cross-sectional area, *bm* body mass, *GJH* Generalized Joint Hypermobility

About one third of the participants had a Beighton score of 9/9, another third had 8/9 and the rest 6 or 7/9. Regarding the Brighton criteria, about 43% fulfilled them and might be diagnosed as having JHS. In addition, 45 of the 51 participants (88.2%) mentioned some pain in the HM-Q at baseline and thus might be diagnosed as having some kind of hypermobility spectrum disorder. The distribution of these participants in both groups was equal (Table 1).

### Primary and secondary outcomes

For both primary outcomes, namely the maximum voluntary strength of knee extensors and flexors, no significant difference was found between the groups when controlling for the baseline difference (ANCOVA *p* = 0.256 for MVC of knee extensors and *p* = 0.365 for MVC of knee flexors) and the effect sizes indicated small effects in favour of the control group (Cohens d = − 0.33 for MVC of knee extensors and - 0.26 for MVC of knee flexors). The secondary outcomes also showed no significant differences between groups with small and heterogeneous effect sizes (Tables 2 and 3).

**Table 2:** Descriptive Data Before and After Training and for the Control Group as Mean Values (Standard Deviation)

	Control Group (n = 24)		Training Group (*n* = 27)	
	Pre	Post	Pre	Post
MVC knee extensors [N/bm]	0.53 (0.14)	0.54 (0.15)	0.63 (0.16)	0.64 (0.17)
RFD knee extensors [N/s/bm]	2.03 (1.31)	1.75 (0.83)	2.70 (1.16)	2.52 (1.23)
MVC knee flexors [N/bm]	0.26 (0.11)	0.29 (0.10)	0.34 (0.12)	0.35 (0.11)
RFD knee flexors [N/s/bm]	0.87 (0.81)	0.74 (0.43)	1.25 (0.90)	0.98 (0.51)
CSA thigh [mm^2^/bm]	24.6 (2.0)	24.4 (1.9)	24.0 (1.6)	24.2 (1.5)
mCSA thigh [mm^2^/bm]	13.1 (2.1)	13.2 (2.1)	13.1 (1.7)	13.3 (1.8)
Muscle mass [mg]	645 (94)	653 (93)	645 (92)	664 (101)
Muscle density [mg/mm^2^]	80.5 (1.8)	80.4 (1.3)	80.8 (1.6)	81.2 (1.2)

*MVC* maximum voluntary contraction strength, *RFD* rate of force development, *CSA* cross sectional area, *mCSA* muscle cross sectional area, *bm* body mass

**Table 3:** Change in Primary and Secondary Variables as Mean Difference and 95% Confidence Interval (CI), Statistical Tests of Group Differences (Including Co-Variates) and Effect Sizes as Cohens d

	Control Group (n = 24)			Training Group (n = 27)			p-value	Effect
	Mean Diff	Lower 95% CI	Upper 95% CI	Mean Diff	Lower 95% CI	Upper 95% CI	^a^	Size^b^
MVC knee extensors [N/bm]	0.011	−0.017	0.038	0.006	−0.034	0.046	0.256	−0.33
RFD knee extensors [N/s/bm]	−0.280	−0.657	0.098	−0.178	−0.563	0.208	0.243	+0.34
MVC knee flexors [N/bm]	0.033	0.002	0.065	0.007	−0.033	0.047	0.365	−0.26
RFD knee flexors [N/s/bm]	−0.125	0.357	0.107	−0.264	−0.522	−0.007	0.689	−0.16
CSA thigh [mm^2^/bm]	−0.18	−0.34	−0.02	0.21	0.02	0.41	0.419	+0.23
mCSA thigh [mm^2^/bm]	0.13	−0.01	0.27	0.22	0.04	0.40	0.169	+0.40
Muscle mass [mg]	7.6	1.8	13.4	19.1	8.3	29.9	0.936	+0.02
Muscle density [mg/mm^2^]	−0.02	−0.42	0.38	0.40	−0.27	1.06	0.131	+0.44

*diff* difference, *MVC* maximum voluntary contraction strength, *RFD* rate of force development, *pSA* cross sectional area, *mCSA* muscle cross sectional area, *bm* body mass

^a^p value for change between groups, using ANCOVA

^b^Effect size between groups as Cohens d: positive values favours resistance training, negative values favours control

### Additional measurements

For additional measurements, the descriptive data are presented in supplementary tables provided in a supporting information file (Luder-G_S3-file_SupportingTables-T4-T7.pdf). For the ground reaction forces all parameters showed no difference in change, indicated by the 95% CI’s which all crossed the zero line (Supporting table T4). In the EMG parameters, the vastus medialis muscle showed a tendency for increased activity during stair descent in the training group, in the control group during descent the vastus lateralis muscle showed a tendency for increased activity, while the biceps femoris muscle tended to decrease during descent. All other comparisons showed no difference, again indicated by the 95% CI’s crossing the zero line (Supporting table T5).

In the various dimensions of the SF-36 no changes related to training or the control period in the control group were seen, only the “social role functioning” for the control group showed a tendency to increase and for “physical functioning” in the training group a tendency to decrease was seen (Supporting table T6). Finally, only the “pain” dimension of the AIMS-2 showed for the training group a tendency to decrease, while no difference was seen in the HM-Q (Supporting table T7).

### Adherence to training and adverse events

The 27 participants in the training group performed a mean of 19.4 (sd 5.3) out of 24 training sessions. 17 women (63.0%) fulfilled more than 20 sessions and thus more than 80% of the program. The mean resistance with one leg on the leg press was 26 kg at the beginning, meaning 42.5% of body mass (sd 24.1). In the last session, the mean resistance was 51 kg and thus 83.5% of body mass (sd 31.5).

In the training group, four participants stopped their training early: Two women due to lack of time, after 5 and 6 sessions respectively. One person stopped after a knee injury not associated with the training and one due to an exacerbation of low back pain, which was classified as an adverse event. Afterwards a lumbar disc hernia was diagnosed and the patient finally underwent surgery. According to the surgeon and an independent physician, it remained unclear if the exacerbation of the pre-existing back problems was activated by the resistance training.

In the control group, 20 of the 24 women took the opportunity to do the resistance training program. However, this was not part of the randomised control trial.

## Discussion

This study evaluated the effects of a 12-week self-guided resistance-training program in women with GJH with and without symptoms. Contrary to our hypothesis, no significant changes in muscle strength or muscle mass compared to the control group were found. Furthermore, the additional functional measurements and questionnaires showed no training-induced changes in daily life function, disability, or pain. This contrasts with several other training studies, i.e. with patients having knee osteoarthritis and performing resistance exercise for the knee muscles. In these studies strength improvements of 15–34% were shown [43], as well as increased muscle cross sectional area of 3–8% [44] and relevant improvements in quality of life and pain [22]. Regarding resistance training in young healthy women strength gains of 20–32% following a 12 week program were demonstrated [45, 46] and improvements in muscle size of 12–17% [47, 48].

When looking at persons with GJH, in three recent studies improvements of muscle strengths were shown, which could not been reproduced in our trial. In 2018 To & Alexander [49] described the same ability to gain strength for persons with JHS, GJH and control subjects by an individualized exercise program, with improvements of about 100% of knee extensor muscle torque in all three groups. Notably, this gain was reached by an exercise program, which was mainly performed home-based with the own body weight, but adaptation and instruction was done every second week by a physiotherapist. Additionally, they found that persons with JHS (according to the Brighton criteria) had about 30% lower muscle torque than controls, while those with GJH (based on the Beighton score) showed about 30% higher torques than controls. These differences in muscle strength might explain the high heterogeneity in our group, since we included both, women with GJH and some with JHS. Liaghat et al. found in 2020 [50] in a feasibility study for heavy shoulder strengthening that participants with HSD (based on Beighton score and a history of shoulder pain) were able to perform a 16-week strength training program and gained about 30% in shoulder strength. In this study the training was performed twice weekly supervised by a physiotherapist and once weekly self-guided. Celenay and Kaya [51] investigated the effect of a 8 week spinal stabilization program performed three time a week in groups and every session guided by a physiotherapist. They found improvements of trunk muscle endurance of about 50%, however their study has some methodological limitations, like a high drop-out rate and missing of blinding. Note, that all these studies were published after the end of our trial and thus the results could not be incorporated in the planning or conduct of our project.

Despite concealed randomisation in our study there was a difference in the baseline parameters between the two groups for maximum strength in knee flexors and extensors. We think, this resulted mainly by chance, since the variability of strength throughout the participants was high. This is indicated by the standard deviations, which are in both cases clearly higher than the mean difference. Furthermore, both parameters were introduced as co-variates in the analysis of covariance. Additional testing without the co-variates did not reveal a difference in the significance testing.

Based on the above-mentioned studies and our own experience during the training sessions, there might be several reasons for the lack of strength and muscle mass improvements in our study. A first problem was the measurement of strength, which was done isometrically, although the training was dynamic. Thus, even when some dynamic strength gain occurred in these participants, this might not have been transferred to the measurement situation and thus could not be detected by our methods. However, if really large changes had occurred, these might be also visible in the isometric contractions, thus we conclude that the dynamic strengths gain, if existing, might be small. Second, many participants exercised at rather low resistance levels. This is illustrated by the fact that the mean resistance during training in the single leg press exercise at the ed. of the 12 weeks was only 83.5% of body mass, with only 8 (of 27) participants using more than their own body weight as training resistance. Third, the training volume with two sessions per week might be not enough, however for persons with little or no experience in resistance training three sessions per week might be quite hard. Additionally, the individual changes due to training showed great variation, which is indicated by the large confidence intervals in both groups. This might be due to the high heterogeneity of the study group, including women fulfilling the Brighton criteria and others who had no symptoms.

In this study the training was mainly performed un-supervised and participants had only three (of 24) sessions with instruction and training adaptation. The last training control and adaptation session was in week six of twelve. Based on the training protocols we recognized that many participants did no longer increase their resistance after week six. Possibly, more guidance during the training and closer supervision of the individual sessions would help these participants to reach the necessary training intensity for a gain in strength and muscle mass. Additionally, a barrier to exercise with higher resistance might have been the fear of an increase in pain or some prior experience of pain reactions after resistance.

The most important limitation of this study might be the heterogeneity of the study group, with some recruits being pre-clinical. Thus, we had participants with few problems and rare pain episodes and others who experienced daily pain and impairments of several daily-life activities. The rather high scores and the large variability in the SF-36 and the AIMS-2, as well as the high standard deviations for the strength and muscle parameters were indications of this. Another indicator for this heterogeneity is the fact that about 43% of the participants fulfilled the Brighton criteria, meaning that we included women with GJH and JHS as well. Recently it was shown that the strength level of these two groups might be quite different [49].

Additional limitations were the outcome measurements. The isometric strength test was difficult to perform for inexperienced participants and possibly did not reflect dynamic changes in muscle strength. In the peripheral quantitative computer tomography, a tendency towards improved muscle density and muscle cross sectional area was seen, but the intensity during the 12 weeks training may not have been high enough to really build up muscle mass. Additionally, nutrition and especially protein intake was not recorded or altered in this study, which might influence the building of new muscle mass [52]. Stair climbing, on the other hand, was an activity that placed minimal demands on the women, so that no changes in their movement patterns were seen. Similarly, the questionnaires showed some ceiling effects, with several participants not having pain or disability in their daily-life. A barrier in this sense was that, at the time of the study, no specific validated questionnaire for hypermobile persons was available. This has now changed, because in 2017 Palmer and colleagues published the Bristol Impact of Hypermobility questionnaire [53]. Then, regarding the intervention, the resistance training program was thoroughly standardised and thus specific adaptations to the individual needs of some of these patients were not possible. In addition, no other interventions were provided, e.g. pain relief techniques or advice on function in daily-life activities. Finally, this study did not incorporate a comparison with women with normal mobility doing the same training program. However, the goal of the project was to compare the strength gain by training in comparison to a control without training in women with GJH, not to compare the strength gains between women with and without GJH. Thus, it was not possible to include two additional groups with women with normal mobility.

One strength of this study was that the program was well suited to the women and the adherence to the training program was good. Several participants mentioned at the end of the program that they intend to continue the resistance training on their own. Only four participants had to stop the training early, three of these for reasons besides the training. In one person with increased low back pain and subsequent lumbar disc hernia, it remained unclear whether the resistance training contributed to this problem.

For future research, it would be of great importance to better define the study group and to include, if possible, mainly asymptomatic persons with GJH. The question of whether these patients can gain muscle mass and strength similar to that of healthy persons has not been finally answered, and more investigation is required as to the best way to guide and monitor such a resistance-training program. Additionally, the training program might be more individualised and could include not just strength exercises but also some functional training or proprioceptive exercises.

## Conclusions

The present study could not identify any effect of a mainly self-guided 12-week resistance training program in women with GJH, compared to a control group. The response to the low intensity resistance training was highly variable and the groups in the study might have been too heterogeneous in terms of symptoms and baseline strength. Possibly, a better guided resistance training program including specific adaptations to the individual needs might be better suitable for these patients. To confine this, more studies with better structure and better suitable training programs are needed.

## Supplementary Information

**Additional file 1: S1.** Detailed description of training program.

**Additional file 2: S2.** Detailed description of the outcome measurements.

**Additional file 3: S3.** Result tables of additional parameters.

**Additional file 4: S4.** Basic dataset supporting the conclusions of this article.

## Data Availability

The basic dataset supporting the conclusions of this article is included within the article as an additional file (Additional file “Luder-G_S4_HM-Training_basic-data-file.xlsx”).

## Abbreviations

AIMS-2: Arthritis Impact Measurement Scales 2
bm: Body mass
BMI: Body mass index
CSA: Cross sectional area
EDS: Ehlers-Danlos syndrome
EMG: Electromyography
GJH: Generalized joint hypermobility
HM-Q: Questionnaire for hypermobility
HSD: Hypermobility spectrum disorder
JHS: Joint hypermobility syndrome
mCSA: Muscle cross-sectional area
MVC: Maximum voluntary contraction strength
pQCT: Peripheral quantitative computer tomography
RFD: Rate of force development
sd: Standard deviation
SF-36: Medical Outcomes Study Short Form
