# Supplementary material for: Effect of resistance training on muscle properties and function in women with generalized joint hypermobility: a single-blind pragmatic randomized controlled trial
Source: BMC Sports Sci Med Rehabil. 2021 Feb 8;13:10. doi: 10.1186/s13102-021-00238-8 (PMC7871640; doi:10.1186/s13102-021-00238-8)
Supplement: Supplementary file 1 — Additional file 1: S1. Detailed description of training program. [file 13102_2021_238_MOESM1_ESM.pdf]

INSELSPITAL
UNIVERSITÄTSSPITAL BERN
HÔPITAL UNIVERSITAIRE DE BERNE

Department of Physiotherapy
Director
Martin L. Verra, PhD, PT

Additional file S1 to **"Effect of Resistance Training on Muscle Properties and Function in Women with Generalized Joint Hypermobility: a Single-Blind Pragmatic Randomized Controlled Trial"**
by Luder Gere, Aeberli Daniel, Mueller Mebes Christine, Haupt-Bertschy Bettina, Baeyens Jean-Pierre and Verra Martin L

Description of the guided 12 week resistance training program

Key training parameters

- Two sessions weekly, resulting in a total of 24 training sessions
- Sessions of about 50 min duration, including 10 minutes warm-up
- First two weeks complex method as introduction: 2 series, 25 repetitions, resistance at 40- 50% of the 1RM, break of at least two minutes between series
- Followed by ten weeks of hypertrophy training: 3 series, 12 repetitions, resistance at 80% of 1RM, break at least 90 sec between series
- Instruction sessions were given in week 1, 3 and 6 by four experienced physiotherapists.
- All other training sessions (up to 21) were performed individually, always with a supervising physiotherapist in the training room.

Providers and place of the intervention

- The training took place in the medical training centre of the Berne University Hospital, Bern, Switzerland. The room is regularly used by the physiotherapists for exercise with patients but is also frequented by former patients or healthy people, who perform their own fitness program. All persons exercising in the fitness room have to undergo extensive instruction and regular control of their exercise. A physiotherapist is always available in the fitness room to instruct and monitor the exercises and to answer any questions the users might have. Most of the users focus mainly on resistance training, but some possibilities for endurance and balance training are also available.
- All machines for the resistance exercise and the warm-up were standard devices for medical use by Technogym (Technogym S.p.A., Cesena, Italy).
- All four providers of the instruction sessions to the study participants were qualified physio-therapists, three of whom had additional training in sports physiotherapy and exercise physiology. The fourth person served as stand-in when the others were not available and only occasionally led instruction sessions.
- Due to the nature of the intervention it was not possible to blind the instruction.
- Detailed documentation of the exercises and the instructions were provided for the physiotherapists. Since all of them also regularly instructed other people (patients and healthy) and took part in the continuous professional development and quality assurance program of all instructors in the medical fitness, no specific training for this study was offered to the physiotherapists. However, one of the study coordinators (GL) was always available for questions.

INSELSPITAL
UNIVERSITÄTSSPITAL BERN
HÔPITAL UNIVERSITAIRE DE BERNE

Department of Physiotherapy
Director
Martin L. Verra, PhD, PT

Description of the guided 12 week resistance training program

Instruction for the resistance training

- All instruction sessions were scheduled by the responsible study coordinator
- Whenever possible all three instruction sessions were delivered by the same physiotherapist.
- The duration of the first instruction session (week 1) was 60 min, the other two sessions (weeks 3 and 6) took about 30 min. Participants were encouraged to be ready in sports clothing at the scheduled time and in session two and three to have done the warm-up before instruction.
- All instructions were performed with one physiotherapist instructing one participant.

First instruction session (60 min)

- Administrative procedure for a new patient (similar as with other persons)
- Administrative procedure for the study: training protocol sheet, box for questionnaires
- Instruction for the warm-up: depending on personal preference the warm-up was done on a cycle ergometer, on a stepper or on a cross-trainer. 10 min of warm-up were recommended, with a low to middle intensity.
- Instruction for the seven resistance exercises: leg press, knee extension and knee flexion while sitting, hip abduction, heel rise on leg press, abdominals and back extension (see below for details and pictures).
- Initial resistance was established by setting a specific weight and asking the person to do as many repetitions as possible. According to the number of possible repetitions the resistance was adapted with the aim of achieving 25 repetitions with 50% of the one repetition maximum (1RM).
- Exercises for the knee and the heel rise were performed unilaterally, doing two series for each side.
- Participants were encouraged to do at least 2 min of break between two series. Exercising the other leg or doing another exercise during the break was allowed but not compulsory.
- Finally the instructions for the notes on the training protocol were given. Actual weight and number of repetitions and series were filled in the specified boxes for each training session.
- Instruction was given to perform three training sessions with this resistance over the next ten days or until the next appointment for the second instruction.

Second and third instruction session (30 min each)

- Check current performance based on the training protocol. Ask if any problems like pain, discomfort or disability had occurred.
- Adapting the resistance for hypertrophy training (second session): Based on the resistance so far the weight was set by the physiotherapist and the number of possible repetitions evaluated. Adaptations were done till about 12 were possible, meaning a resistance of about 80% of the 1RM.
- Further increase of resistance in week 6 (third instruction session) and encouragement to further increase the resistance, whenever more than 12 repetitions were possible.
- Check the notes on the training protocol

All other sessions

- The remaining 21 sessions were performed individually according to the instructions.
- Participants were encouraged to schedule the two sessions per week with at least two days between.
- In the training room a responsible physiotherapist was always present for support and to answer questions. Depending on daytime the room is used by 5 to 30 persons in parallel.

INSELSPITAL
UNIVERSITÄTSSPITAL BERN
HÔPITAL UNIVERSITAIRE DE BERNE

Department of Physiotherapy
Director
Martin L. Verra, PhD, PT

Description of the guided 12 week resistance training program

Adherence and adverse events

- As already mentioned all participants had an individual training protocol for the documentation. From these the adherence to the training program was evaluated, whereby more than 80% of the permitted training sessions (i.e. 21 sessions) was rated as acceptable performance.
- Additionally, all participants filled in a short weekly questionnaire for pain and disability, including two questions about side-effects of the training or problems occurring after the training sessions.

Description of exercises

Finally, the seven exercises are illustrated here as performed by a participant. The starting and the end positions on the device and the relevant instructions for joint angles and range of motion (ROM) are shown for each exercise.

Starting position | **End position**

Exercise 1: Single leg squat on leg-press, for thigh muscles, sitting position with approximately 80° of hip flexion. ROM knee from 100° flexion to 5° flexion. Performed with both legs individually.

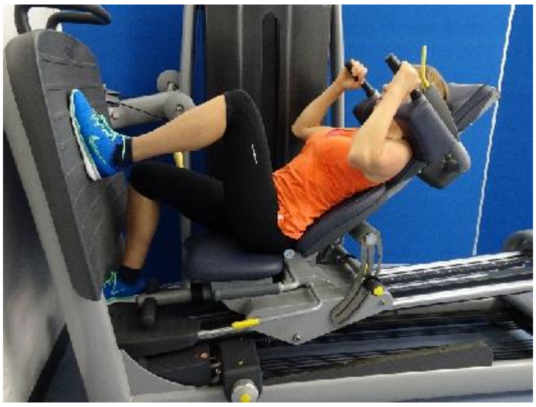

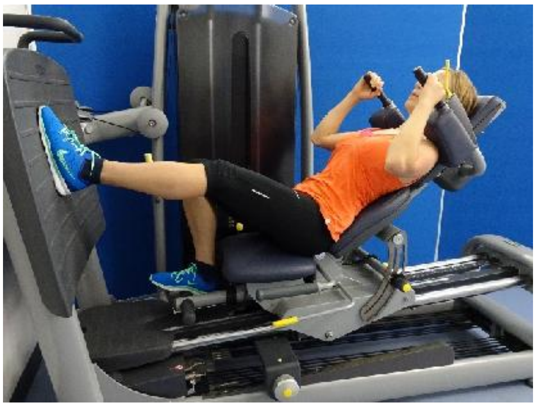

Exercise 2: Single leg knee extension, resis-tance at ankle ,for quadriceps muscle, sitting position, approximately 120° of hip flexion. ROM knee from 130° of flexion to 10° flexion. Performed with both legs individually.

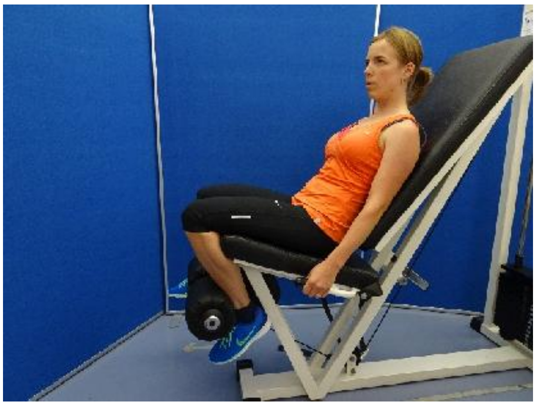

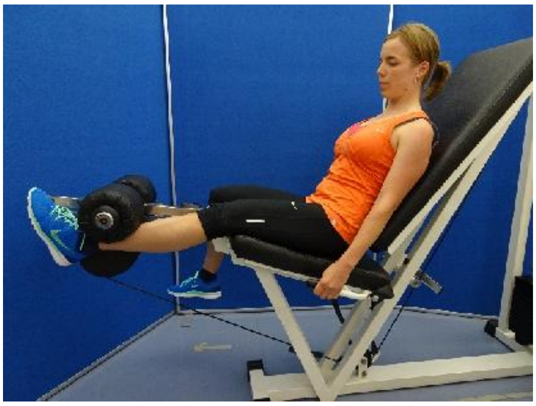

Exercise 3: Single leg knee flexion, with resistance at ankle, for hamstring muscles, standing on opposite leg, trunk lean forward approximately 40° in the hip. Range of knee motion from 10° of flexion to 100° flexion. Performed with both legs individually.

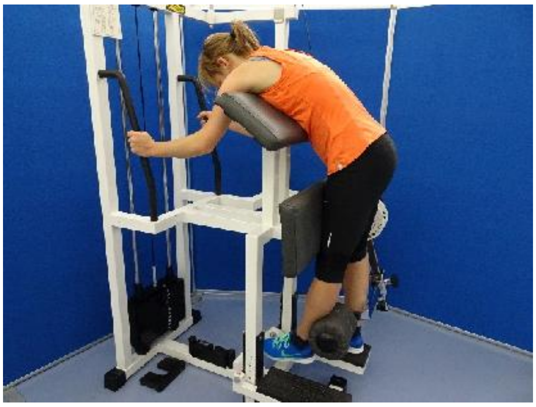

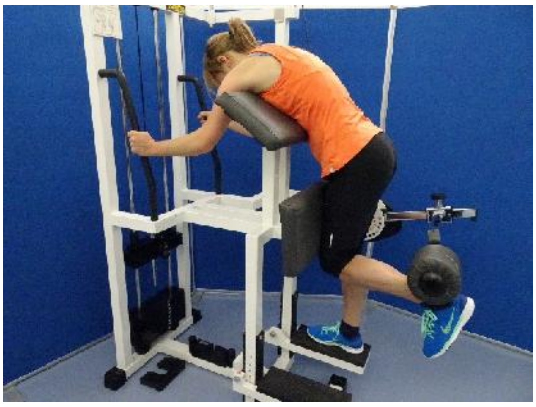

The depicted model gave consent to publish the pictures in scientific journals

INSELSPITAL
UNIVERSITÄTSSPITAL BERN
HÔPITAL UNIVERSITAIRE DE BERNE

Department of Physiotherapy
Director
Martin L. Verra, PhD, PT

Description of the guided 12 week resistance training program

Starting position | **End position**

Exercise 4: Bilateral hip abduction in sitting position, resistance laterally at the lower half of the thigh, hip range of motion from 10° to 40° of abduction.

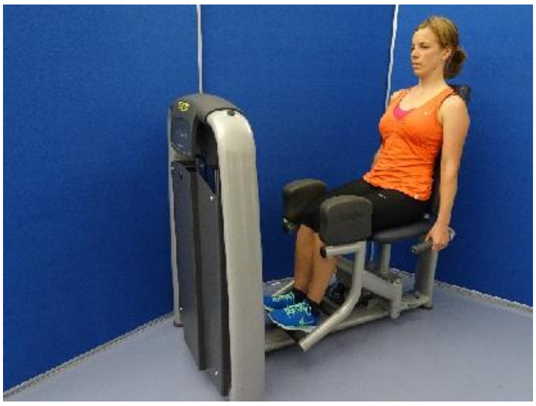

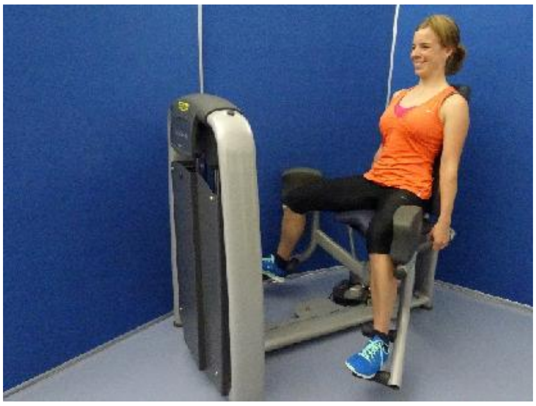

Exercise 5: Single leg heel rise on leg press for triceps surae, sitting position with approximately 80° of hip flexion and 90° of knee flexion. Performed with both legs individually.

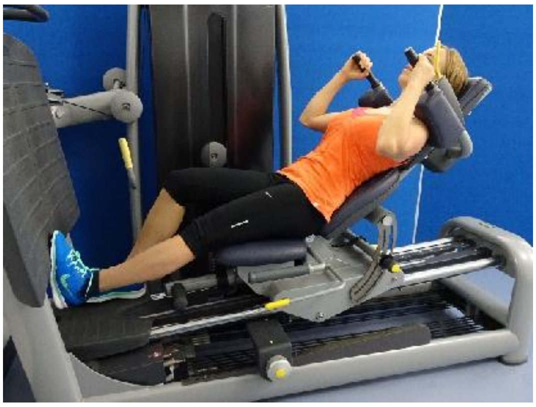

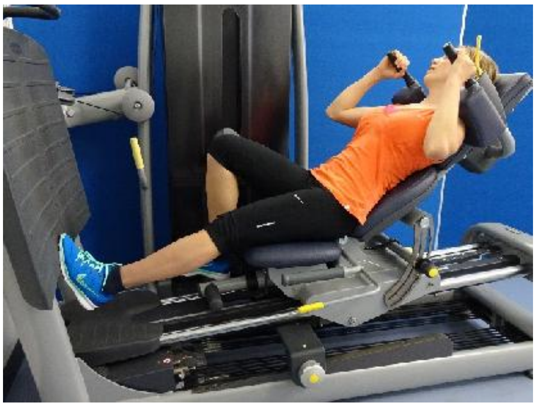

Exercise 6: Back extension, resistance bilaterally at shoulder plates, sitting position. Range of hip motion from 120° to 80° of hip flexion.

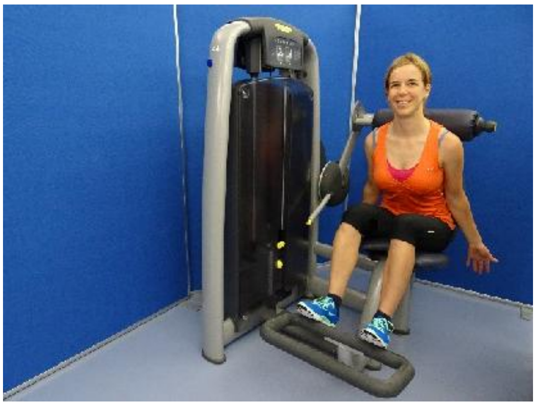

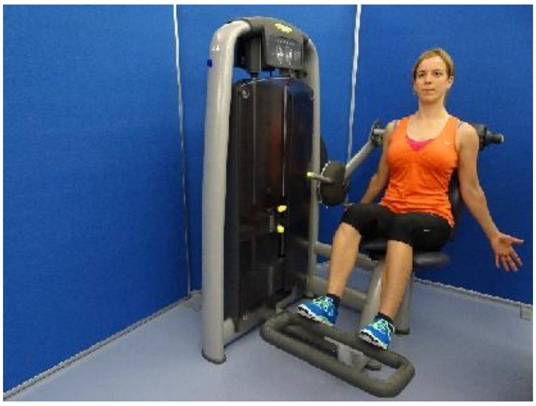

Exercise 7: Abdominals, resistance at chest in a sitting position.

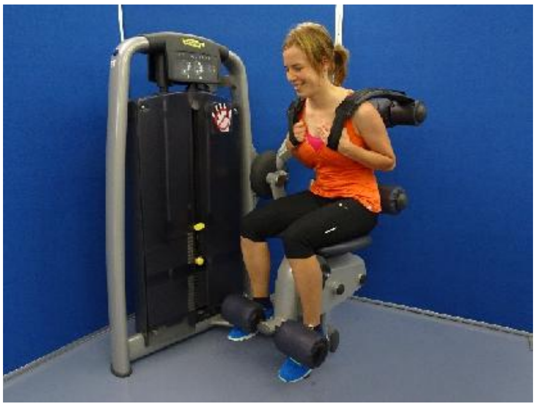

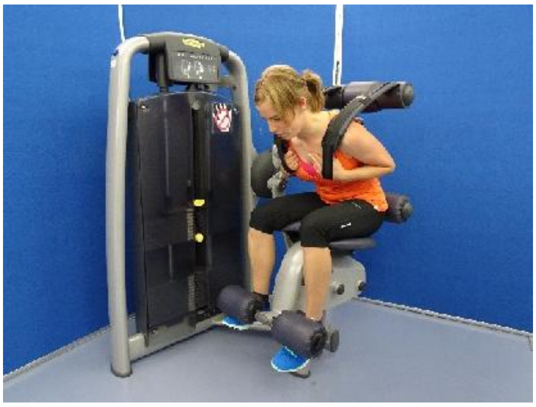

The depicted model gave consent to publish the pictures in scientific journals
