# Supplementary material for: Effect of resistance training on muscle properties and function in women with generalized joint hypermobility: a single-blind pragmatic randomized controlled trial
Source: BMC Sports Sci Med Rehabil. 2021 Feb 8;13:10. doi: 10.1186/s13102-021-00238-8 (PMC7871640; doi:10.1186/s13102-021-00238-8)
Supplement: Supplementary file 2 — Additional file 2: S2. Detailed description of the outcome measurements. [file 13102_2021_238_MOESM2_ESM.pdf]

# Additional File S2 to “Effect of Resistance Training on Muscle Properties and Function in Women with Generalized Joint Hypermobility: a Single-Blind Pragmatic Randomized Controlled Trial”

by Luder Gere, Aeberli Daniel, Mueller Mebes Christine, Haupt-Bertschy Bettina, Baeyens Jean-Pierre, Verra Martin L.

## Detailed description of the outcome assessments

Musculoskeletal disorders may affect an individual in several dimensions of life. According to the World Health Organisation (WHO) the impact of health problems is described on four levels: body functions, body structures, activities and participation, and environmental factors as defined in the International Classification of Functioning, Disability and Health (ICF) (World Health Organisation, 2001). The outcome assessments in this study aimed to evaluate the effects of a strength training program in women with GJH in all dimensions of the ICF. In terms of body structure muscle strength was measured isometrically and muscle and bone properties by peripheral quantitative computer tomography (pQCT). Regarding body functioning, muscle activity and ground reaction force during stair climbing were measured as usual activities related to movement control. Stair climbing was also included to assess the dimension of activities and participation, and patient reported questionnaires were used to detect impairments in daily life. Some environmental factors, mainly in terms of relationship and attitudes, were included in the questionnaires. Since there was no specific questionnaire for persons with GJH available, a set of three questionnaires was used to get wide-ranging information on activities, participation and environmental factors. In detail, the following assessments with their respective analyses were used in the study:

**Muscle strength:** The maximum isometric strength (MVC) and the rate of force development (RFD) of the knee extensors and knee flexors were measured on a custom-built strength measurement table with a one-dimensional strain gauge (KM 1500S; Megatron, Munich, Germany) calibrated in Newton (N). The participant sat with 90° of knee and hip flexion on the table and above the ankle a sling, connected to the force transducer, was tightly attached. The participant was asked to push forward as fast and as strong as possible and to hold the highest possible force for five seconds for the measurement of extensor strength. Then the force transducer was changed to the ventral attachment and the participant was asked to pull backwards in the same way for the flexors. After familiarization and two test trials, the three measuring trials were performed with a break in between of 30 s. During strength testing the EMG of the thigh muscles was recorded in sync with the respective force signals of the transducer (as described below).

To analyse muscle strength the maximum voluntary contraction (MVC), i.e. the maximal force in Newton and rate of force development (RFD) defined as the slope of the force curve between 20% and 80% of MVC in Newton/second, were calculated for knee extensors and knee flexors. For MVC and RFD the values were normalised to body mass and the maximal value of the three trials was taken.

**Muscle properties:** Using a Stratec XCT 3000 scanner (Stratec Medizintechnik, Pforzheim, Germany) the muscle cross sectional area (mCSA) and the muscle mass and density at the thigh as well as the total area of the thigh were measured at 33% of femur length above the knee by peripheral quantitative computer tomography (pQCT), as previously described (Aeberli et al., 2010).

For the analysis, the muscle variables were calculated with the integrated software of the device. Main variables were the mCSA and the total CSA in $mm^2$ as well as the muscle mass in

mg and the muscle density in $mg/cm^3$. Both CSA parameters were calculated in relation to body mass.

**Muscle activity:** The muscle activity during stair climbing of four muscles of the right leg was measured using surface EMG: vastus medialis (VM) and vastus lateralis (VL) as knee extensors and semitendinosus (ST) and biceps femoris (BF) as knee flexors. Electrode placement and measurement procedure were defined according to the recommendations of ISEK (Merletti & Torino, 1997) and SENIAM (Hermens, 2000). In detail, after marking the correct electrode positions the skin was prepared by shaving and cleaning. For each muscle two oval pre-gelled AgCl-electrodes (Ambu Blue Sensor N, Ambu A/S, Ballerup, Denmark) of 5 mm diameter were placed in parallel 2 cm apart. Additionally, a single reference electrode was placed laterally over the femoral condyle. Skin impedance for each pair of electrodes was measured by an impedance meter (Digitimer D175, Digitimer Ltd, Welwyn Garden City, UK) and had to be below 5 kΩ. Otherwise, the electrodes were removed and the procedure with shaving and cleaning was repeated. All electrodes were then connected by cable via pre-amplifiers (baseline noise<1uV RMS, input impedance>100MΩ, common mode rejection ratio>100dB, input range of +/- 10mV, base gain of 500, and a 10-500Hz bandpass filter) to a small telemetry box (TeleMyo 2400T G2, Noraxon, Scottsdale, Arizona, USA), which was worn on the participant’s back. From there the signals were transmitted to a receiver (TeleMyo 2400R G2, Noraxon, Scottsdale, Arizona, USA) and recorded at a sampling rate of 1 kHz using a 12-bit analog-digital converter (Meilhaus ME-2600i, SisNova Engineering, Zug, Switzerland) and the software package “ads” (version 1.12, uk-labs, Kempen, Germany).

**Stair climbing**: To measure ground reaction forces (GRF) and EMG during stair ascent and descent a custom-built wooden six-step stair-case was used (riser height 17.9 cm, tread 29 cm, inclination 30.4°, according to Stacoff et al. (Stacoff, Diezi, Luder, Stüssi, & Kramers-De Quervain, 2005)). The stair has a handrail on both sides for security reasons and ends with a platform of one meter length to allow for comfortable turning. All steps were covered with non-slippery mats. GRFs were measured using two force plates (Type 9286BA, Kistler Winterthur, Switzerland) embedded in the 3rd and 4th step of the staircase and supported by an independent heavy-weight steel frame. The vertical force signals of both force plates were transmitted via a custom-built amplifier (uk-labs, Kempen, Germany) to the recording computer. To determine the second foot contact of the stride, which was not measured on a force plate, a tri-axial accelerometer (Model 317A, Noraxon, Scottsdale, Arizona, USA) was attached to the right malleolus and connected to the EMG telemetry system as described above. These signals were then recorded in sync with the EMG and the GRF and registered together in the software package “ads” (version 1.12, uk-labs, Kempen, Germany). The participants had to climb up and down the six steps ten times at a comfortable, self-selected speed barefoot and without using the handrail.

All EMG and GRF data were processed using a custom-made MATLAB toolbox (The MathWorks, Natick, Massachusetts, USA) in accordance with previously described algorithms(Luder et al., 2015; Stacoff et al., 2005). The measurements were visually inspected and six trials selected for separate analyses of stair ascent and descent in accordance with existing recommendations (Shiavi, Frigo, & Pedotti, 1998). The EMG of the MVC measurements served as a basis for normalization calculated by RMS over 500ms using the highest value out of three trials. Dynamic EMG data was baseline corrected, fully rectified and normalised to the corresponding 100% MVC value and linear envelopes built by low pass-filtering (second-order Butterworth, cut-off 20 Hz (Hug, 2011)). Peak muscle activation during stance was calculated from the linear envelopes. The vertical force–time curves were low-pass filtered (second-order Butterworth, cut-off 30 Hz), normalised to each participant’s body mass and parameterised according to previously described standard methods (Stacoff et al., 2005). Foot contact and foot off

were defined as the time points when the vertical force exceeded or fell below 3% of the subject’s body mass, respectively. Foot contact at the end of the stride was determined by visual analysis of the raw accelerometer signal. The maximum force-peak during weight acceptance (Fmax) was calculated as well as the respective time after the starting point (t to Fmax) and the slope of the force curve during the loading phase (loading rate). The mean value of six trials for each subject and condition was calculated for all parameters.

**General health:** The Medical Outcomes Study Short Form 36-Item (SF-36) was used to measure general health status of the participants. The SF-36 is a widely used multi-item generic health survey, which is available in German. The psychometric properties are well established and normative values for many patient groups are available, including those in the field of rheumatology (Busija et al., 2011). The SF-36 was used in the first clinical trial looking at the effects of a home program on the proprioception of hypermobile persons(Ferrell et al., 2004). The questionnaire is self-administered and takes about 10 minutes to complete. The questions target eight physical and mental health domains like physical, psychological and social functioning (Busija et al., 2011). The SF-36 scores were calculated according to the standard method (Busija et al., 2011), resulting in scores for all subscales, each ranging from 0-100, with higher values indicating better health-related quality of life, and two additional sum scores.

**Disability in daily life:** Since there was no specific questionnaire for persons with GJH available at the time of the study preparation, the Arthritis Impact Measurement Scales 2 (AIMS-2) was chosen to evaluate disability in daily life and restrictions in participation. The AIMS-2 is an assessment of physical functioning, pain, psychological status and other domains, originally developed for patients with rheumatoid arthritis and osteoarthritis. The questionnaire has a total of 78 questions and takes about 20 minutes to complete (Gignac, Cao, McAlpine, & Badley, 2011). The AIMS-2 is available in German and the psychometric properties are well established (Rosemann & Szecsenyi, 2007). The scale was also used in one of the published clinical trials with hypermobile persons (Sahin et al., 2008). The scores for the AIMS-2 were calculated according to the described methods (Gignac et al., 2011; Rosemann & Szecsenyi, 2007), resulting in a total score of between 0- 10, with high scores indicating poor health. Scores for seven subscales (walking and bending. social activity, pain, level of tension, satisfaction, health perceptions, impact) and three component models (physical component, affect, social interaction) were calculated.

**Hypermobility questionnaire (HM-Q):** A simple self-developed and face-validated questionnaire for pain and disability was also used. Participants were asked to provide information about their current pain intensity and locations on five-point Likert scales. The HM-Q had 28 items, of which 16 targeted pain in different body regions and 12 questions asked about disability in daily activities like bending, stair climbing, sitting for more than one hour or carrying loads. A sum score was calculated and scaled between 20-100 with lower values indicating better health. The activities in the questionnaire were chosen based on the most frequently mentioned problem situations in a previous cross-sectional study (Mueller Mebes et al., 2018).

### Conduct of assessments

All assessments were performed by a single investigator (GL), blinded to group allocation. The first assessment took place before the training or control period and the second after the end of the training or after the 12 week waiting period. The second assessment had to take place within two weeks after the last training session. Completion of all assessments took about two hours for every participant at every time point.

## References

Aeberli, D., Eser, P., Bonel, H., Widmer, J., Caliezi, G., Varisco, P. A., … Villiger, P. M. (2010). Reduced trabecular bone mineral density and cortical thickness accompanied by increased outer bone circumference in metacarpal bone of rheumatoid arthritis patients: A cross-sectional study. *Arthritis Research and Therapy*, *12*(3), R119. https://doi.org/10.1186/ar3056

Busija, L., Pausenberger, E., Haines, T. P., Haymes, S., Buchbinder, R., & Osborne, R. H. (2011). Adult measures of general health and health-related quality of life. *Arthritis Care & Research*, *63*(S11), S383–S412. https://doi.org/10.1002/acr.20541

Ferrell, W. R., Tennant, N., Sturrock, R. D., Ashton, L., Creed, G., Brydson, G., & Rafferty, D. (2004). Amelioration of symptoms by enhancement of proprioception in patients with joint hypermobility syndrome. *Arthritis and Rheumatism*, *50*(10), 3323–3328. https://doi.org/10.1002/art.20582

Gignac, M. A., Cao, X., McAlpine, J., & Badley, E. M. (2011). Measures of disability. *Arthritis Care and Research*, *63*(S11), S308–S324. https://doi.org/10.1002/acr.20640

Hermens, H. J. (2000). Development of recommendations for SEMG sensors and sensor placement procedures. *Journal of Electromyography and Kinesiology*, *10*, 361–374. https://doi.org/10.1016/s1050-6411(00)00027-4

Hug, F. (2011). Can muscle coordination be precisely studied by surface electromyography? *Journal of Electromyography and Kinesiology*, *21*(1), 1–12. https://doi.org/10.1016/j.jelekin.2010.08.009

Luder, G., Schmid, S., Stettler, M., Mueller Mebes, C., Stutz, U., Ziswiler, H. R., & Radlinger, L. (2015). Stair climbing - An insight and comparison between women with and without joint hypermobility: A descriptive study. *Journal of Electromyography and Kinesiology*, *25*(1), 161–167. https://doi.org/10.1016/j.jelekin.2014.07.005

Merletti, A. R., & Torino, P. (1997). Standards for reporting EMG data. *Journal of Electromyography and Kinesiology*, *7*(2), I–II. https://doi.org/10.1016/S1050-6411(97)90001-8

Mueller Mebes, C., Luder, G., Schmid, S., Stettler, M., Stutz, U., & Radlinger, L. (2018). Symptoms in Daily Life and Activity Level of Women with and without. *Rheumatology: Current Research*, *8*(3), 1–7. https://doi.org/10.4172/2161-1149.1000241

Rosemann, T., & Szecsenyi, J. (2007). Cultural adaptation and validation of a German version of the Arthritis Impact Measurement Scales (AIMS2). *Osteoarthritis and Cartilage / OARS, Osteoarthritis Research Society*, *15*(10), 1128–1133. https://doi.org/10.1016/j.joca.2007.03.021

Sahin, N., Baskent, A., Cakmak, A., Salli, A., Ugurlu, H., & Berker, E. (2008). Evaluation of knee proprioception and effects of proprioception exercise in patients with benign joint hypermobility syndrome. *Rheumatology International*, *28*(10), 995–1000. https://doi.org/10.1007/s00296-008-0566-z

Shiavi, R., Frigo, C., & Pedotti, A. (1998). Electromyographic signals during gait: Criteria for envelope filtering and number of strides. *Medical and Biological Engineering and Computing*, *36*(2), 171–178. https://doi.org/10.1007/BF02510739

Stacoff, A., Diezi, C., Luder, G., Stüssi, E., & Kramers-De Quervain, I. A. (2005). Ground reaction forces on stairs: Effects of stair inclination and age. *Gait and Posture*, *21*(1), 24–38. https://doi.org/10.1016/j.gaitpost.2003.11.003

World Health Organisation. (2001). *International classification of functioning, disability, and health : ICF*. Geneva: World Health Organization.
