# Supplementary material for: Effect of resistance training on muscle properties and function in women with generalized joint hypermobility: a single-blind pragmatic randomized controlled trial
Source: BMC Sports Sci Med Rehabil. 2021 Feb 8;13:10. doi: 10.1186/s13102-021-00238-8 (PMC7871640; doi:10.1186/s13102-021-00238-8)
Supplement: Supplementary file 3 — Additional file 3: S3. Result tables of additional parameters. [file 13102_2021_238_MOESM3_ESM.pdf]

Supporting Table T4. Descriptive Data of Parameters from the Vertical Ground Reaction Force During Stair Ascent and Descent Before and After Training and for the Control Group as Mean Values (Standard Deviation) and the Respective Changes as Mean Difference and 95% Confidence Interval (CI)

	Control Group (n = 24)					Training Group (n = 27)				
	Pre	Post	Mean Diff	95%CI Lower	95% CI Upper	Pre	Post	Mean Diff	95%CI Lower	95% CI Upper
Fmax Up [%bm]	109.2 (6.6)	108.9 (6.8)	-0.3	-1.6	1.0	108.9 (6.1)	107.8 (6.4)	-1.0	-2.4	0.4
t to Fmax Up [ms]	200.3 (31.4)	199.1 (27.4)	-1.1	-7.3	5.0	205.9 (29.8)	208.5 (24.4)	2.5	-6.0	11.1
Loading Rate Up [%bm/s]	122.6 (50.2)	124.4 (44.9)	1.8	-4.2	7.8	113.9 (34.1)	111.1 (30.6)	-2.8	-9.3	3.7
Contact Time Up [ms]	734.5 (89.6)	726.6 (78.5)	-7.9	-26.3	10.5	764.3 (85.1)	768.2 (73.9)	3.9	-20.6	28.5
Fmax Down [%bm]	141.4 (13.7)	142.5 (12.5)	1.1	-2.8	8.0	140.0 (13.0)	141.5 (13.8)	1.5	-2.3	5.4
t to Fmax Down [ms]	157.6 (20.5)	159.5 (17.7)	1.9	-3.9	7.6	169.7 (21.1)	168.2 (17.2)	-1.5	-8.9	5.9
Loading Rate Down [%bm/s]	170.8 (54.9)	170.2 (53.2)	-0.7	-9.5	8.2	154.0 (37.0)	153.3 (37.1)	-0.8	-9.8	8.3
Contact Time Down [ms]	701.4 (104.2)	694.4 (85.7)	-7.0	-27.4	13.4	730.3 (84.0)	731.0 (65.1)	0.9	-26.4	27.7

diff = difference, Fmax = maximal force during weight acceptance, bm = body mass, t = time

Supplorting Table T5. Descriptive Data of Maximal Muscle Activation Derived by Electromyography (EMG) During Stair Ascent and Descent Before and After Training and for the Control Group as Mean Values (Standard Deviation) and the Respective Changes as Mean Difference and 95% Confidence Interval (CI)

EMG Muscle [% MVC]	Control Group (n = 24)					Training Group (n = 27)				
	Pre	Post	Mean Diff	95%CI Lower	95% CI Upper	Pre	Post	Mean Diff	95%CI Lower	95% CI Upper
Biceps Femoris max up	16.6 (18.8)	17.9 (18.4)	1.26	-1.87	4.40	10.8 (9.2)	9.9 (7.9)	-0.89	-2.41	0.64
Semitendinosus max up	17.5 (16.0)	17.6 (19.1)	0.10	-2.17	2.36	14.1 (11.3)	11.5 (9.9)	-2.57	-5.41	0.26
Vastus Lateralis max up	44.8 (24.1)	47.8 (25.6)	2.93	-5.21	11.07	36.5 (19.2)	40.7 (24.6)	4.21	-2.34	10.75
Vastus Medialis max up	45.2 (31.6)	45.5 (37.5)	0.34	-9.84	10.52	36.5 (20.1)	48.5 (35.7)	11.95	-0.43	24.32
Biceps Femoris max down	9.7 (11.8)	7.7 (9.5)	-1.93	-3.29	-0.57	5.4 (3.9)	4.6 (3.7)	-0.76	-1.99	0.47
Semitendinosus max down	12.4 (13.6)	9.5 (10.4)	-2.97	-6.69	0.76	6.8 (6.0)	5.7 (6.0)	-1.02	-2.65	0.61
Vastus Lateralis max down	25.8 (17.6)	34.4 (30.3)	8.55	1.46	15.64	20.0 (9.6)	21.4 (17.6)	1.49	-2.56	5.55
Vastus Medialis max down	25.9 (16.9)	29.0 (25.1)	3.08	-2.12	8.27	20.3 (10.6)	27.8 (12.3)	7.56	0.83	14.24

MVC = maximal voluntary contraction

Supporting Table T6. Descriptive Data for the Eight Dimensions of the SF-36 Before and After training and for the Control Group as Mean Values (Standard Deviation) and the Respective Changes, as Mean Difference and 95% Confidence Interval (CI)

Dimension of SF-36	Control Group (n = 24)					Training Group (n = 27)				
	Pre	Post	Mean Diff	95%CI Lower	95% CI Upper	Pre	Post	Mean Diff	95%CI Lower	95% CI Upper
Physical Functioning	95.0 (11.3)	94.8 (10.1)	-0.21	-1.37	0.95	94.1 (9.9)	91.5 (12.0)	-2.59	-4.74	-0.44
Physical Role Functioning	89.6 (25.5)	91.7 (25.2)	2.08	-2.23	6.39	90.1 (24.5)	87.0 (27.2)	-3.09	-9.99	3.83
Bodily Pain	84.8 (24.2)	83.1 (20.0)	-1.67	-8.97	5.63	75.3 (18.2)	77.1 (20.4)	1.74	-3.76	7.25
General Health Perception	79.8 (16.4)	82.5 (13.5)	2.63	-1.32	6.57	75.9 (22.5)	74.1 (23.3)	-1.85	-4.56	0.85
Vitality	58.8 (19.1)	60.0 (17.4)	1.25	-3.12	5.62	53.2 (21.0)	54.3 (22.0)	1.05	-3.52	5.62
Social Role Functioning	87.0 (16.3)	90.1 (13.3)	3.13	0.32	5.93	93.1 (13.6)	92.6 (15.2)	-0.46	-5.86	4.93
Emotional Role Functioning	90.3 (20.1)	95.8 (15.0)	5.56	-1.22	12.33	95.1 (12.1)	84.0 (26.8)	-11.11	-22.68	0.45
Mental Health	73.5 (17.4)	76.5 (14.7)	3.00	-1.52	7.52	73.3 (20.8)	75.4 (19.2)	2.11	-3.31	7.54
Physical Health (sum score)	54.6 (7.3)	54.2 (7.1)	-0.41	-1.93	1.11	52.3 (7.1)	52.0 (7.9)	-0.36	-2.04	1.31
Mental Health (sum score)	49.2 (8.7)	51.2 (8.1)	2.05	-0.01	4.11	51.3 (6.8)	50.8 (8.2)	-0.54	-3.95	2.87

Scoring for individual dimensions is 0-100 with lower scores indicating poorer health, for the sum scores 50 is equivalent to the US-norm population values.

Supporting Table T7. Descriptive Data for the Dimensions of the AIMS-2 and the Hypermobility Questionnaire Before and After training and for the Control Group as Mean Values (Standard Deviation) and the Respective Changes, as Mean Difference and 95% Confidence Interval (CI)

Dimensions of AIMS-2	Control Group (n = 24)					Training Group (n = 27)				
	Pre	Post	Mean Diff	95%CI Lower	95% CI Upper	Pre	Post	Mean Diff	95%CI Lower	95% CI Upper
Walking, Bending	0.4 (0.8)	0.5 (1.2)	0.04	-0.23	0.31	0.6 (0.8)	0.8 (1.7)	0.19	-0.50	0.87
Social Activity	4.4 (1.3)	4.2 (1.4)	-0.20	-0.62	0.22	4.3 (2.0)	3.8 (2.0)	-0.48	-0.97	0.01
Pain	1.3 (1.8)	1.3 (1.7)	0.00	-0.27	0.27	1.7 (1.9)	1.3 (1.7)	-0.39	-0.75	-0.03
Level of Tension	3.4 (1.8)	3.3 (1.4)	-0.06	-0.70	0.57	3.5 (1.3)	3.4 (1.7)	-0.09	-0.62	0.43
Satisfaction	0.9 (1.5)	0.7 (0.7)	-0.24	-0.72	0.25	0.9 (1.4)	1.1 (1.2)	0.16	-0.14	0.46
Health Perceptions	2.6 (2.2)	2.6 (2.0)	0.00	-0.72	0.72	3.1 (2.3)	3.1 (2.1)	0.00	-0.64	0.64
Impact	1.9 (1.9)	2.0 (2.2)	0.12	-0.55	0.79	2.1 (2.4)	2.1 (2.4)	0.00	-0.48	0.48
Physical Component	0.3 (0.7)	0.2 (0.5)	-0.06	-0.18	0.06	0.2 (0.3)	0.2 (0.4	0.02	-0.10	0.13
Affect	2.7 (1.6)	2.6 (1.3)	-0.07	-0.65	0.50	2.8 (1.2)	2.8 (1.4)	0.04	-0.32	0.40
Social Interaction	3.2 (1.0)	3.2 (1.1)	0.01	-0.36	0.37	3.1 (1.3)	3.0 (1.1)	-0.17	-0.42	0.09
HM-Q (sum score)	29.0 (8.6)	27.1 (7.0)	-1.82	-3.73	0.08	32.6 (10.9)	31.1 (7.9)	-1.59	-3.82	0.64

AIMS-2 = Arthritis impact measurement scales 2 (scale 0-10, lower scores indicating better health), HM-Q = Hypermobility questionnaire (scale 20-100, with lower values indicating better health)
